# Cognitive Ability in Late Adolescence and Disability Pension in Middle Age: Follow-Up of a National Cohort of Swedish Males

**DOI:** 10.1371/journal.pone.0078268

**Published:** 2013-10-16

**Authors:** Alma Sörberg, Andreas Lundin, Peter Allebeck, Bo Melin, Daniel Falkstedt, Tomas Hemmingsson

**Affiliations:** 1 Institute of Environmental Medicine, Karolinska Institutet, Stockholm, Sweden; 2 Department of Public Health Sciences, Karolinska Institutet, Stockholm, Sweden; 3 Division of Psychology, Department of Clinical Neuroscience, Karolinska Institutet, Stockholm, Sweden; 4 Centre for Social Research on Alcohol and Drugs, Stockholm University,Stockholm, Sweden; University of Adelaide, Australia

## Abstract

Low cognitive ability in late adolescence has previously been shown to be associated with disability pension (DP) in young adulthood. However, most DP’s are granted later in working life, and the mechanisms of the association are not fully understood. We aimed to investigate the association between cognitive ability in late adolescence and DP at ages 40-59, and investigate the role of individual and socioeconomic factors. Information on cognitive ability, health status, personality aspects and health behaviours at age 18-20 was obtained from the 1969-70 conscription cohort, comprising 49 321 Swedish men. Data on DP’s 1991-2008 was obtained from the Longitudinal Database of Education, Income and Employment. Information on socioeconomic and work-related factors in childhood and adulthood was obtained from national sociodemographic databases. Hazard ratios for DP during follow-up were estimated by Cox proportional hazards models. We found a graded relationship between cognitive ability in late adolescence and DP in middle age. One step decrease on the nine-point stanine scale of cognitive ability was associated with a crude hazard ratio of 1.26 (95 % CI 1.24-1.27). Socioeconomic and work-related circumstances in adulthood explained much of the association, but factors measured already in late adolescence also showed importance. The findings suggest an accumulation of risks over the life course. Although attenuated, the graded relationship remained after adjusting for all factors.

## Introduction

In Sweden, as in most European countries, disability pension (DP) is a part of the welfare system that ensures subsistence living for people who are unable to work due to long-standing illness or injury. According to the National Insurance Act, DP may be granted to adults up to age 65 whose working capacity is judged to be permanently reduced [1]. In most cases, it provides full-time compensation and implies a permanent exclusion from the labour market. About 8% of the Swedish population aged 16-64 (i. e., up to the regular retirement age of 65) received a DP in 2010, and the proportion increases with age [2]. Mental illness and musculoskeletal diseases account for a majority of DP’s among both women and men [2]. While comparisons between countries on DP-related benefits is a complex issue, Sweden is among the countries with the highest prevalence and largest public spending on DP, and the number of newly granted DP’s has increased greatly during the last two decades [3]. In the mid-2000’s, regulations were tightened but is still a highly debated issue on political level. As well as being of economic concern to society, DP may lead to a social and economic decline for the individual [4]. Determinants of DP are found at societal, work-related and individual levels [5]. 

Cognitive ability has gained attention as a predictor for various health outcomes in many longitudinal studies. Lower cognitive ability, measured in childhood or adolescence, is associated with various health outcomes, including somatic and psychiatric morbidity [6,7] as well as mortality [8]. In longitudinal studies of large conscription cohorts (men only), lower cognitive ability in adolescence has been associated with higher risk of DP in early adulthood, i. e., up to age 43 [9,10]. In those studies, the associations were attenuated but remained significant after adjustments for social background, mental health, education and other factors. However, adult socioeconomic position was controlled for in just one of these studies, which adjusted for occupation at age 24 [9]. 

Although it has been suggested that education contributes to increased cognitive ability [11], it is also likely that cognitive ability determines educational attainment [12]. Thus, it is possible that lower cognitive ability leads to a higher risk of DP via the mediums of education and occupation; fewer years of education have been shown to be associated with DP [13]. People with higher cognitive ability tend to reach higher socioeconomic positions [14], which in turn are associated with less adverse physical and psychosocial working conditions [15] and a lower proportion of granted DP’s [16]. Cognitive ability is also a determinant of other labour market outcomes, such as long-term sickness absence and unemployment [17,18]. Since these conditions are themselves risk factors for DP [19], it is conceivable that they lie on the pathway leading to permanent exclusion from the labour market. 

Cognitive ability has also been shown to be associated with health behaviours early in life such as alcohol consumption and smoking [20], which in turn have shown to be risk factors for DP [21,22]. The risk factors associated with cognitive ability that lead to later DP may already be detectable in late adolescence, before entry into the labour market. There may also be mediating effects of socioeconomic circumstances and labour market experiences in adulthood. Individual factors, measured prior to labour market entry, and attained education and occupation have not previously been investigated together in relation to the association between cognitive ability and DP. Further, the association between cognitive ability and DP has not been studied beyond 43 years of age, although most DP’s are granted at ages older than that.

The aim of this study was to investigate the association between cognitive ability and DP in middle age. Specifically, we wanted to find out: (1) whether there indeed was an association between cognitive ability and DP; (2) if so, the extent to which it was explained by socioeconomic, psychosocial and health-related factors measured prior to labour market entry; and (3) the extent to which it was mediated by factors related to socioeconomic position, labour market attachment and work characteristics in adulthood.

We used the Swedish conscription cohort of 1969-70, which consists of 49 231 men born in 1949-51. From the conscription examination, data is available on conditions in childhood and adolescence, including tests of cognitive ability. Record linkage with national sociodemographic databases provided information about socioeconomic and labour market factors in childhood and adulthood as well as data on DP during follow-up.

## Methods

### Ethics statement

Ethical approval was obtained from Stockholm’s Regional Ethical Review Board at Karolinska Institutet, decision reference number 2004/5:9- 639/5. Due to the character of the database and the anonymization of all data, the Review Board waived the normal requirement for written consent.

### Study population

The study was based on data from a nationwide examination of 49 321 young Swedish males aged 18-20, who were conscripted for compulsory military service in 1969 and 1970. The background to the Swedish conscription examination and the variables it measured have been presented in detail elsewhere [23–25]. Only 2-3% of all Swedish men were exempted from conscription at that time, in most cases due to severe handicaps or congenital disorders. Ninety-eight per cent of all men who were conscripted in 1969 and 1970 were born in 1949-1951; the remaining 2% were born before 1949, and are excluded from the study in order to increase homogeneity. Data collected at different time points, and obtained from various databases and registers (see Table 1), were combined using unique identification codes. The database and record linkage data are anonymized. 

**Table 1:** Timing of data collection.

Information obtained	Year(s)	Age	
Birth year	1949-51	0	
Childhood socioeconomic position (occupation of head of household)	1960	9-11	The National Population and Housing Census of 1960
Cognitive ability, self-reported smoking and alcohol use, BMI; psychologists’ ratings of personality, clinical diagnoses of psychiatric and musculoskeletal disorders.	1969-70	18-20	Conscription examination
Unemployment (number of years with any unemployment benefit 1974-90)	1974-90	23-41	The Income and Tax Register
Education (years attained), income	1990	39-41	The Longitudinal Database of Education, Income and Employment (LOUISE)
Socioeconomic position in adulthood (occupation)	1990	39-41	The National Population and Housing Census of 1990
Job control and physical strain, by occupation	1990	39-41	Job exposure classification systems and LOUISE
Sickness absence 1990-91 (number of days), unemployment 1992-94 (number of days with benefit)	1991-94	40-45	LOUISE
Disability pension (first award)	1991-2008	40-59	LOUISE

### Exposure: Cognitive ability

Cognitive ability was assessed at conscription in 1969-70 by four subtests: a verbal test (synonym detection), a logical-inductive test (comprehension of written instructions), a visuospatial test (paper form board test of geometric perception) and a test of technical comprehension (mechanical and physics problems). The cognitive assessment has been described in detail elsewhere [25–27]. All four tests were progressive, starting with relatively easy questions and then increasing in difficulty. The results were converted to normally distributed standard-nine (stanine) scales for each subtest, with scores 1-9. These were then combined and transformed into a new stanine scale as a global measure of general ability, corresponding to approximate IQ bands of: < 74, 74–81, 82–89, 90- 95, 96–104, 105–110, 111–118, 119–126, > 126. Of the men, 49 262 (99.9%) had a score on cognitive ability [27]. 

### Outcome: Disability pension

Information on granted DP 1991-2008, at ages 40-59, was obtained from the Longitudinal Database of Education, Income and Employment (Swedish acronym LOUISE), administered by Statistics Sweden [28]. Newly granted DP’s were added to the LOUISE database at the end of each year. In Sweden, DP is granted by the National Social Insurance Agency to people with a permanently reduced work capacity due to medically certified illness or injury. 

### Covariates

#### Childhood socioeconomic position

Information on socioeconomic position at ages about 9-11 was obtained from the National Population and Housing Census of 1960. Socioeconomic position was based on the occupation of the head of household, most often the father. Occupations were classified into six socioeconomic groups: (1) unskilled workers; (2) skilled workers; (3) assistant non-manual employees; (4) non-manual employees at intermediate or higher level; (5) farmers; (6) those not classified into any other socioeconomic group. 

#### Covariates collected at the conscription examination

Besides the cognitive ability assessment described above, all the men underwent a medical examination and a structured interview with a trained psychologist during the two-day conscription process. Height and weight were recorded and used for calculating body mass index (BMI, kg/m^2^). Any medical diagnoses were recorded according to the ICD-8. In any case where psychiatric illness was reported or suspected, the conscript was referred to a psychiatrist who, if appropriate, made a diagnosis, also according to the ICD-8.

A psychologist assessed personality aspects by following detailed guidelines, and giving ratings on a scale from one to five. A low rating of “emotional control” was given to conscripts who seemed to lack the ability to control nervousness and anxiousness, or to channel aggression, and/or had documented lower functional capacity for psychosomatic reasons. A high rating was given to conscripts who seemed able to act calmly in most kinds of situations. Low emotional control has previously been associated with DP up to 43 years of age in this cohort [9]. A low rating of “social maturity” was given to conscripts who seemed dependent on others, or irresponsible, and/or showed signs of social maladjustment. A high rating was given to those who seemed willing to take responsibility and who showed signs of independence, dominance and extroversion. Low social maturity has been associated with various adverse health-related outcomes in this cohort [29,30]. Psychologists’ ratings were regularly checked for inter-rater reliability [31]. 

 All the men completed questionnaires regarding social and psychosocial adjustment, psychological symptoms, and substance use. Smoking was placed into categories of no smoking, and smoking 1-10, 11-20 and >20 cigarettes per day. A composite variable, “risky use of alcohol”, was constructed from affirmative answers to one or more of the following: consumption of >250 g 100% alcohol/week, reporting the drinking of an ‘eye-opener’ during a hangover, having been apprehended for drunkenness, or having ‘often’ been drunk (with other choices ‘rather often’, ‘sometimes’, and ‘never’). This variable is associated with DP up to age 59 in this cohort [21].

#### Socioeconomic and work-related factors in adulthood

Information on education, unemployment after 1990, income and sickness absence benefits was obtained from the LOUISE database, described above. Information on unemployment before 1990 was obtained from the Income and Tax Register. Education was categorized according to the highest education obtained by 1990, measured in years: ≤9, 10-11, 12-13, 14 and ≥15 years. Total number of years with any unemployment benefit between 1976 and 1990 was used as a measure of stability on the labour market, and categorized into five groups: 0, 1-2, 3-5, 6-9 and >9 years in total. Further, unemployment during the recession in Sweden in 1992-1994 was categorized into three groups of zero, 1-90 or > 90 days of unemployment benefit during this period, as described previously [32]. Income in 1990 was calculated as quartiles of income from employment, business and unemployment benefit. Number of days on sick leave in 1990 -1991 was calculated from the proportion of total income derived from sickness benefit, and then converted into quintiles of the total number of days on sick leave.

Information was obtained from the National Population and Housing Census of 1990 regarding socioeconomic position at 39-41 years of age. The classification into eight socioeconomic groups was based on occupation: (1) unskilled workers; (2) skilled workers; (3) assistant non-manual employees; (4) non-manual employees at intermediate level; (5) non-manual employees at higher level; (6) farmers; (7) self-employed (mostly skilled workers or drivers); (8) those for whom no occupation was reported. 

Classification of level of job control and physical strain at work was made using exposure classification systems, based on the Swedish Work Environment Surveys and the Swedish Annual Level of Living Surveys. The procedure is described in greater detail by Johansson et al. [13], who have previously shown these variables to be associated with DP in this cohort. The variables were presumed to provide more precise measures of job-related risk factors within occupational levels. Job control and physical strain at work were divided into quartiles, from highest to lowest. 

### Statistical analyses

Hazards ratios (HR) for DP during the period 1991-2008 in relation to level of cognitive ability at conscription were estimated by the Cox proportional hazards model using SAS 9.2. Cognitive ability was modeled as a continuous variable, with HR’s given for each one-step decrease on the stanine scale, and also for each score on the stanine scale, with score five (5) as reference. Proportionality was assessed in log-log survival plots. We investigated the effects of adjusting for the variables separately, and then for two sets of combined variables. The first set included factors collected before or soon after presumed entry into the labour market, i.e., up to and including conscription; and the second included factors in adulthood, when most men in the sample were presumed to have entered the labour market. In contrast to factors in the first set, those in the second set were registered several years after the cognitive assessment that had been conducted at conscription, and were regarded as possible mediators. Next, we divided follow-up into two time periods, 1991-1999 and 2000-2008, and repeated the analyses in crude and adjusted models. We also conducted an analysis adding the exposure classifications of job control and physical workload in a subsample of men with information on these factors. Subsequently, we conducted an analysis to adjust for sickness absence in 1991-92 and unemployment in 1993-94, with follow-up 1995-2008. These adjustments were made in order to investigate the possible mediating role of exclusion from the labour market during the recession of the early 1990s in the association between cognitive ability and DP.

## Results

Among the 44 144 men alive at baseline, and with information on all the variables included in the analyses, 4 899 were granted a DP during the entire follow-up period, 1991-2008. Men in the study sample were similar to the full cohort with regard to background factors in childhood and at conscription (data available on request).

**Figure 1:**
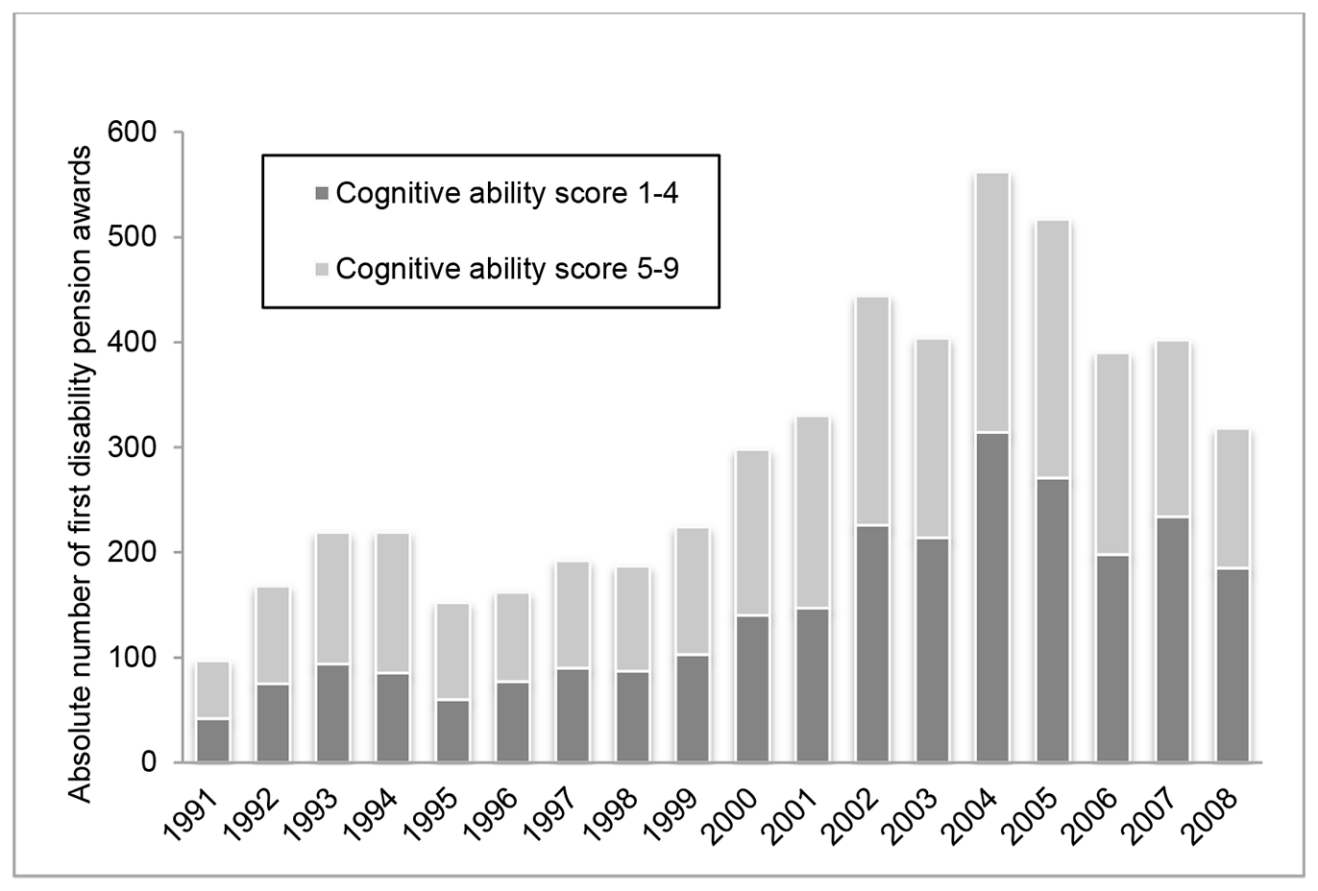
Disability pension 1991-2008 by cognitive ability. Number of men receiving a first award of disability pension 1991-2008, and among them the proportions of men obtaining cognitive ability scores 1-4 and 5-9 on the stanine scale. In the full cohort, 33% scored 1-4 on the cognitive ability test.

Figure 1 shows the number of men who were granted a DP between 1991 and 2008 by year, and among them the proportion of men with a stanine score of less than 5 on cognitive ability. The number of disability pension awards increased with age until 2004 and then declined; this is in line with the national trend, which shows a peak followed by a steep decline in the number of newly granted DP’s during the 2000s [2]. About 33% of the men in the full cohort scored below 5, while the corresponding proportion among those who received a DP is 52% (not shown in the figure).

Table 2 shows the associations between covariates and DP. Factors across the life course, from childhood socioeconomic position and various factors measured at conscription to work factors in middle age, were found to be associated with subsequent DP up to age 59. A psychiatric diagnosis at conscription, being unemployed 1974-1990 or during the economic recession of the early 1990s, having more sickness absence days than most men in the early 1990s, and having a manual occupation (compared with men in non-manual occupations) were the strongest predictors of DP.

**Table 2:** Associations between covariates and disability pension 1991-2008, in crude hazard ratios (HR’s) with 95% confidence intervals.

		**Disability pension 1991-2008**	
**Risk factor**	No. exposed	**HR**	**CI 95%**
**Pre labour market entry**			
Low childhood socioeconomic position (manual occupation^a^)	24 125	1.48	1.39-1.58
Low emotional control	12 826	1.99	1.88-2.11
Low social maturity	9423	1.97	1.85-2.09
Smoker (≥1 cigarette/day)	25 713	1.64	1.54-1.74
Risky use of alcohol	5656	1.94	1.81-2.08
BMI≥25 (overweight/obese)	2896	1.43	1.29-1.57
Psychiatric diagnosis at conscription	4578	2.46	2.29-2.63
Musculoskeletal diagnosis at conscription	7366	1.33	1.24-1.43
**Adulthood, post labour market entry**			
Unemployment 1974-1990 (> 0 days)	12 875	2.23	2.11-2.36
Low educational level (≤9 yrs), 1990	11 327	1.68	1.58-1.78
Low income (lowest quintile), 1990	10 288	1.61	1.52-1.70
Manual occupation, 1990^a^	16 468	2.42	2.26-2.60
Low job control (lowest quartile), 1990^b^	9797	1.87	1.75-1.99
High physical strain at work (highest quartile), 1990^b^	11 307	1.49	1.39-1.59
Sickness absence 1990-91 (highest two quintiles, 8-528 days)^c^	16 370	3.39	3.18-3.62
Unemployment 1992-94 (>0 days)^c^	8176	2.41	2.26-2.57

^a^ For the purpose of obtaining a single measure of the relative risk of DP associated with socioeconomic position (SEP), the HR’s of manual occupation (compared with non-manual occupation) as reference were analyzed in subsamples of 38 268 (childhood SEP) and 36 078 (adult SEP). In all the main analyses, adjustments were made for all the occupational groups.

^b^ Job exposure variables were analyzed in a subsample of 39 714 men.

^c^ Sickness absence 1990-91 and unemployment 1992-94 were analyzed in a subsample of 42 234 men with follow-up starting in 1995.

Table 3 shows the association between cognitive ability at conscription and DP 1991-2008. The association is measured as a crude HR, and as HR’s after adjustment for the factors separately, and in models that combine factors measured before and after presumed labour market entry. The socioeconomic factors reported in 1990 had the strongest impacts on the association, with a risk reduction of 23-27% for each of the separate adjustments. Factors measured at conscription also had some attenuating impacts on the association, primarily the personality aspects assessed by a psychologist, self-reported health lifestyle factors, and psychiatric diagnoses. Musculoskeletal diagnoses at conscription and socioeconomic position in childhood had only minimal impacts. 

**Table 3:** Relative risk (hazard ratio, HR, with 95% confidence interval) of the granting of disability pension 1991-2008 per one (1) point decrease in cognitive ability score at conscription on a stanine scale, with adjustments made for each variable separately and in combinations of variables according to stage of life.

	**Disability pension 1991-2008**		
*N= 44 144; 4 899 disability pensions granted.*			
	**HR**	**CI 95%**	**Reduction in HR (%)**
**Crude**	**1.26**	**1.24-1.27**	
**Adjusted for:**			
A. Pre labour market entry			
Childhood SEP	1.25	1.23-1.27	4
Personality factors (emotional control, social maturity)	1.21	1.19-1.22	19
Health lifestyle factors (smoking, risky use of alcohol, high BMI score)	1.23	1.21-1.25	12
Psychiatric diagnosis at conscription	1.23	1.21-1.24	12
Musculoskeletal diagnosis at conscription	1.25	1.24-1.27	4
**All factors pre labour market entry**	**1.19**	**1.17-1.21**	**27**
B. Adulthood, post labour market entry			
Unemployment 1974-90 (years with benefit, 5 groups)	1.22	1.20-1.23	15
Education	1.19	1.17-1.21	27
Adulthood BMI	1.20	1.18-1.21	23
Income	1.19	1.17-1.20	27
**All adult factors, post labour market entry**	**1.13**	**1.11-1.15**	**50**
**A + B: Full adjustment**	**1.11**	**1.09-1.13**	**58**

**Figure 2:**
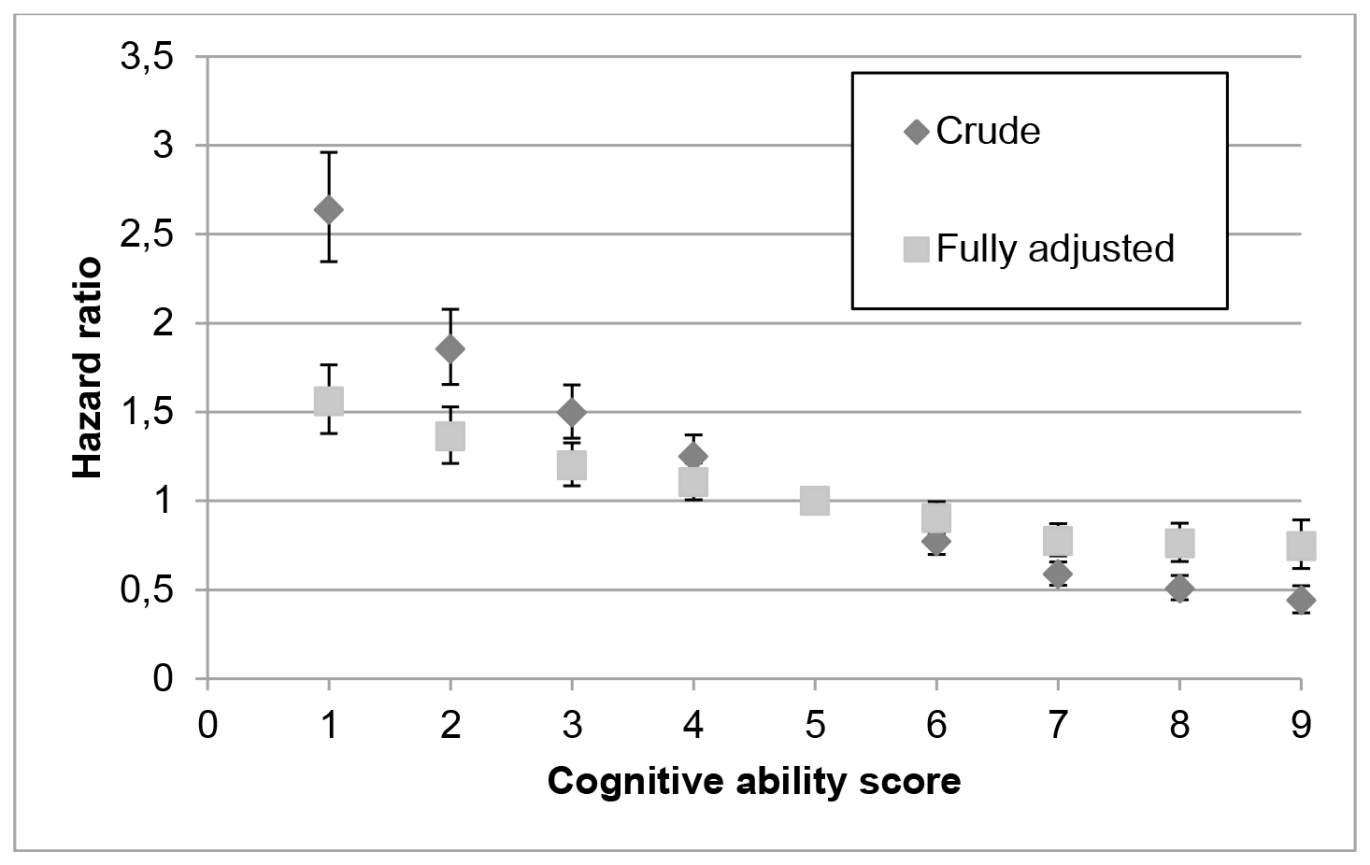
Association between cognitive ability and disability pension. Relative risks (hazard ratios, HR’s, with 95% confidence intervals) of the granting of disability pension 1991-2008 by cognitive ability score at conscription on a stanine scale, in crude (unadjusted) and fully adjusted models, with a cognitive ability score of five as reference. Adjustments are made for childhood socioeconomic position, personality aspects, health-related lifestyle factors (smoking, risky use of alcohol, BMI score), psychiatric and musculoskeletal diagnoses, unemployment, education, attained socioeconomic position, and income.

Adjusting for all factors collected in childhood and at conscription, i.e., before most of the men entered the labour market, yielded a risk reduction of 27%. In comparison, adjusting for factors related to attained socioeconomic position, labour market attachment, and social factors in adulthood reduced risk by 50%. Adjusting for all factors from childhood to middle age reduced risk by 58%. The association between cognitive ability and DP remained statistically significant in the fully adjusted model. It was apparent across the stanine scale in both the unadjusted and fully adjusted models (Figure 2).

Adjusting for job control and physical workload, in a subsample of 39 714 men with complete data from the job exposure classification system in 1990, had no impact on the estimate, over and above the effects of all other factors (crude HR 1.25, 95% CI 1.23-1.27; adjusted HR 1.11, 1.09-1.13 in models including or excluding job exposure variables). In another subsample of 42 234 men with follow-up starting in 1995, adjusting for number of sickness days 1990-91 and number of unemployment days 1992-1994 had only a small impact on the estimate, over and above the effects of the other factors (crude HR 1.24, 1.09-1.13; HR 1.11, 1.09-1.13 adjusted for all other factors; HR 1.09, 1.07-1.11 including sickness absence and unemployment).

Table 4 shows that the crude HR for DP 1991-1999 is of greater magnitude than for DP during the later follow-up 2000-2008 (HR’s 1.32, 95% CI 1.29-1.36, and 1.23, 95% CI 1.21-1.35, respectively), but the difference decreases when factors measured across the life span are added to the models. The association between cognitive ability at conscription and disability pension remained statistically significant in the fully adjusted model for both time periods.

**Table 4:** Associations between cognitive ability at conscription 1969-70 and disability pension 1991-1999/2000-2008, in crude and adjusted hazard ratios (HR’s), with 95% confidence intervals, per one (1) point decrease in cognitive ability score on a stanine scale.

	**Disability pension 1991-1999**			**Disability pension 2000-2008**		
	*N=44 144, 1480 DP’s granted*			*N=42 045,3419 DP’s granted*		
	**HR**	**CI 95%**	**Reduction in HR (%)**	**HR**	**CI 95%**	**Reduction in HR (%)**
**Crude**	**1.32**	**1.29-1.36**		**1.23**	**1.21-1.25**	
**Adjusted for:**						
Pre labour market entry factors ^a^	1.23	1.20-1.27	28	1.17	1.15-1.19	26
Adulthood, post labour market entry factors^b^	1.16	1.13-1.20	50	1.11	1.09-1.13	52
**Full adjustment^c^**	**1.13**	**1.10-1.17**	**59**	**1.09**	**1.07-1.11**	**61**

^a^ Childhood socioeconomic position, personality aspects, health-related lifestyle factors (smoking, risky use of alcohol, high BMI score) and psychiatric and musculoskeletal diagnoses at conscription.

^b^ Unemployment, education, attained socioeconomic position, income.

^c^ All of the above.

## Discussion

In this cohort study, covering nearly all Swedish men eligible for mandatory conscription in 1969-70, we found that lower cognitive ability at conscription was associated with disability pension between age about 40 and 59. This was evident across the full range of cognitive ability. The magnitude of the association was reduced by 58% when various factors across the life course were added to the model, including socioeconomic position in childhood, personality, health behaviours, psychiatric and musculoskeletal diagnoses in late adolescence, and attained education and socioeconomic position in middle age. Nevertheless, the association remained statistically significant after full adjustment.

### Comparison with previous studies

Low cognitive ability has been found to be associated with DP, at earlier ages than shown here, in previous studies of Scandinavian conscripts [9,10]. In these studies, cognitive ability was one of many factors in multivariate analyses and the association between cognitive ability and DP was not adjusted for any other factors. An earlier study on this cohort found an increased risk of DP up to age 43 among men with a score less than five on the stanine scale, which was attenuated but remained statistically significant after adjustment for various background factors and socioeconomic position at age 24 [9]. Here, we showed that the higher risk associated with lower cognitive ability remained among men in their 40s and 50s, even after adjustments for several covariates measured up to age 40. We also found that the association was not limited to individuals scoring below average, but occurred across the full scale of cognitive ability. Further, socioeconomic and work-related factors proved to be more important than early-life factors in explaining the association of cognitive ability with DP in middle age. 

Henderson et al. [18] investigated associations between cognitive ability and similar outcomes, namely self-reports of being permanently sick or disabled and receiving incapacity benefit, in three British cohorts. In line with our findings, controlling for education and adult social class partly reduced the increased risks associated with lower cognitive ability, but social class in childhood and a history of depression had smaller or no effects on the associations.

Among the variables measured at conscription, emotional control and social maturity, as evaluated by psychologists, had the greatest attenuating impacts. These variables have previously been shown to explain some of the association between cognitive ability and suicidal behavior in this cohort [29]. Associations between cognitive ability and personality have previously been reported, but the mechanisms are complex and not fully understood [33]. Results from twin studies suggest that cognitive ability and personality traits share genetic bases [34]. Similar to cognitive ability, personality has been shown to be stable over time and an important determinant of health outcomes [35]. More specifically, neuroticism, a personality trait, has been found to be a risk factor for DP due to low back pain in a Finnish longitudinal study [36]. Childhood socioeconomic position was found to have only a marginal attenuating impact on the association. This is in line with previous studies of cognitive ability and other health outcomes in this cohort [20]. 

### Interpretation of findings

The link between cognitive ability and health, and its underlying mechanisms, have been investigated in several studies [8]. Some possible explanations, which are not mutually exclusive, have been presented [37]: 1. Living conditions and complications during early development can influence both cognitive ability and health later in life, thus potentially confounding the association; 2. Cognitive ability predicts educational attainment and social position, which in turn have an impact on health outcomes and therefore act as mediators in the association; 3. Both cognitive ability and health may reflect the individual’s innate physiological composition, where higher ‘body system integrity’ is regarded as beneficial to health; 4. Cognitive ability may be associated with health management ability, e.g., to understand health messages and to take appropriate self-care actions. These suggested explanations are likely to vary according to the specific health outcome considered.

The substantial attenuating effects of socioeconomic factors at age about 40 on the association between early cognitive ability and later DP suggest that they act as mediators in the relationship. Moreover, individual factors measured in late adolescence, before any major effects of factors in the adult social environment on health would have been possible, was found to explain about a quarter of the magnitude of the association. These factors capture both early life factors and health management (e g smoking and alcohol use) in the models above. They may also capture physiological composition, although adjustment for musculoskeletal disorders at conscription had almost no effect on the association. The additional effect of factors in adulthood, once earlier factors were taken into account, is likely to reflect an accumulation of risk over time. Risk factors for poor health recorded at conscription are more prevalent among men in lower socioeconomic positions in adulthood, and have previously been found to give rise to socioeconomic differences in health [38]. 

Also, previous studies based on the same cohort have found that the association between cognitive ability and cardiovascular disease, which is an important cause of DP among middle aged men [2], disappears after adjustment for factors similar to the ones considered in this study [27]. By contrast, in the current study, a substantial part of the association between cognitive ability and DP was found to remain after full adjustment. One explanation might be that the major reasons for DP’s, i.e., psychiatric and musculoskeletal diagnoses, are associated with early cognitive ability via different mechanisms than cardiovascular disease. By comparison, the association between cognitive ability and suicidal behavior, which is closely related to other psychiatric conditions, also remained significant in this cohort after adjustment for various factors, including attained socioeconomic position [29]. However, associations between cognitive ability and long-term sickness absence in British cohorts were only minimally affected by adjustment for depression, indicating that psychological distress is not crucial to the association [18]. Another explanation might be that cognitive ability is a resource in case of illness, as yet another expression of the suggested health management mechanism described above. Henderson et al. [18] suggest that people with higher cognitive ability might be better at getting appropriate support from health professionals, managers or colleagues as they need it. The authors also suggest that people with lower cognitive ability or less education may have greater difficulty in transferring skills and finding a job they can manage in case of reduced work capacity. 

Although the association declined in magnitude from the 1990s to the 2000s, a significant association remained even after about 50 years of age. By comparison, the association between cognitive ability and later mental and physical health was found to prevail from middle age to well above retirement age in the Whitehall II cohort [26]. The persistence of the association can partly be explained by the consistency of cognitive ability over the life course, as shown by Deary et al. [39] among others. An accumulation of risk and health damage over time, as mentioned above, may also contribute to the persistent association. Nevertheless, the magnitude of the association decreased between the 1990s and the 2000s, which may be related to the great increase in the number of DP’s in the early 2000s. It is likely that the factors that are less strongly related to cognitive ability become more important with increasing age.

Although we could control for a large range of covariates, factors not measured in this study might further explain the association. For example, an adverse psychosocial environment in childhood may hamper cognitive development [40], and also increase the risk of future DP [41]. Gravseth et al. [10] suggest that coping and mastery might be mediators in the individual realm. Assessments of emotional control and social maturity may capture some aspects of coping and mastery skills, but are not exact measures of them. Further, cognitive ability has been found to correlate with job satisfaction [42,43], which, in turn, may be a protective factor for DP [44,45]. One explanation could be that higher cognitive ability is related to higher education, and thereby better job opportunities. Indeed, we found that DP was more common among men with low educational level and less advantageous job circumstances (high physical strain and low control).

### Strengths and limitations

The cohort is large and highly representative of Swedish men born around 1950. Information from mandatory conscription in late adolescence included the results of cognitive testing using a standardized test battery and of a full medical examination. The unique identification numbers used in Sweden allowed us to link conscription data to reliable national registers with high coverage, up to 39 years after conscription. However, only men were included and our findings cannot be generalized to women, particularly since risk factors for DP have been shown to differ between the sexes [19,45].

Health-related lifestyle factors were measured at one time point only, and changes in health behaviours over time might lead to misclassification. For example, smoking cessation among men who smoked at conscription was common; however, smoking cessation was not significantly related to cognitive ability [46]. Moreover, since the data on health, behaviours and childhood socioeconomic position were collected during childhood and adolescence, rather than retrospectively, the risk of recall bias is reduced. 

## Conclusion

In this longitudinal cohort study, cognitive ability in late adolescence was found to be inversley associated with DP between the ages of 40 and 59. The association was present across the full range of cognitive ability. Adjusting for a number of socioeconomic work-related and personality factors attenuated the association considerably. However, the association remained statistically significant even after full adjustment.
