# Correction: Cognitive Ability in Late Adolescence and Disability Pension in Middle Age: Follow-Up of a National Cohort of Swedish Males

**DOI:** 10.1371/journal.pone.0103291

**Published:** 2014-07-21

**Authors:** The PLOS ONE Staff

There is an error in Table 3. The line “Adulthood BMI” should read “Adulthood SEP”. The corrected table is shown below.

**Table 3:** Relative risk (hazard ratio, HR, with 95% confidence interval) of the granting of disability pension 1991-2008 per one (1) point decrease in cognitive ability score at conscription on a stanine scale, with adjustments made for each variable separately and in combinations of variables according to stage of life.

	Disability pension 1991-2008		
*N = 44 144; 4 899 disability pensions granted.*			
	HR	CI 95%	Reduction in HR (%)
**Crude**	**1.26**	**1.24-1.27**	
**Adjusted for:**			
A. Pre labour market entry			
Childhood SEP	1.25	1.23-1.27	4
Personality factors (emotional control, social maturity)	1.21	1.19-1.22	19
Health lifestyle factors (smoking, risky use of alcohol, high BMI score)	1.23	1.21-1.25	12
Psychiatric diagnosis at conscription	1.23	1.21-1.24	12
Musculoskeletal diagnosis at conscription	1.25	1.24-1.27	4
**All factors pre labour market entry**	**1.19**	**1.17-1.21**	**27**
B. Adulthood, post labour market entry			
Unemployment 1974-90 (years with benefit, 5 groups)	1.22	1.20-1.23	15
Education	1.19	1.17-1.21	27
Adulthood SEP	1.20	1.18-1.21	23
Income	1.19	1.17-1.20	27
**All adult factors, post labour market entry**	**1.13**	**1.11-1.15**	**50**
**A + B: Full adjustment**	**1.11**	**1.09-1.13**	**58**
